# Ensemble Learning Based on Policy Optimization Neural Networks for Capability Assessment

**DOI:** 10.3390/s21175802

**Published:** 2021-08-28

**Authors:** Feng Zhang, Jiang Li, Ye Wang, Lihong Guo, Dongyan Wu, Hao Wu, Hongwei Zhao

**Affiliations:** 1 Changchun Institute of Optics, Fine Mechanics and Physics, Chinese Academy of Sciences, No. 3888, Dongnanhu Rd., Changchun 130033, China; zf00401@outlook.com (F.Z.); wangyeciomp@outlook.com (Y.W.); guolh@ciomp.ac.cn (L.G.); 2 University of Chinese Academy of Sciences, No. 19, Yuquan Rd., Beijing 100049, China; 3 School of Aviation Operations and Services, Aviation University of Air Force, No. 2222, Dongnanhu Rd., Changchun 130022, China; wdy0924@outlook.com; 4 PLA96901 Unit, No. 109, Beiqing Street, Haidian District, Beijing 100094, China; wh340323814@outlook.com; 5 School of Computer Science, Jilin University, No. 2699, Qianjing Rd., Changchun 130012, China; Zhaohw@jlu.edu.cn

**Keywords:** capability assessment, policy optimization, ensemble learning, artificial neural network, index sensitivity

## Abstract

Capability assessment plays a crucial role in the demonstration and construction of equipment. To improve the accuracy and stability of capability assessment, we study the neural network learning algorithms in the field of capability assessment and index sensitivity. Aiming at the problem of overfitting and parameter optimization in neural network learning, the paper proposes an improved machine learning algorithm—the Ensemble Learning Based on Policy Optimization Neural Networks (ELPONN) with the policy optimization and ensemble learning. This algorithm presents an optimized neural network learning algorithm through different strategies evolution, and builds an ensemble learning model of multi-intelligent algorithms to assess the capability and analyze the sensitivity of the indexes. Through the assessment of capabilities, the algorithm effectively avoids parameter optimization from entering the minimum point in performance to improve the accuracy of equipment capability assessment, which is significantly better than previous neural network assessment methods. The experimental results show that the mean relative error is 4.10%, which is better than BP, GABP, and early stopping. The ELPONN algorithm has better accuracy and stability performance, and meets the requirements of capability assessment.

## 1. Introduction

Capability assessment plays a crucial role in the development and construction of equipment. Through the assessment of the inherent capabilities of equipment, the relationship between key elements of indexes and equipment capabilities is sought, and the intelligent assessment method of equipment capabilities based on the equipment index system in operational use is studied. In order to solve the problem of capability assessment, researchers have proposed a variety of solutions, including fuzzy comprehensive assessment method, analytic hierarchy process, principal component analysis method, gray correlation method, operational simulation method, among others. In recent years, the application and development of artificial intelligence algorithms such as neural networks, reinforcement learning, and evolutionary learning have attracted increasing attention in this field.

The assessment of equipment capability has developed rapidly in recent years. Davis [1] of RAND Corporation proposed an exploratory analysis method, and gave a multi-resolution multi-perspective modeling (MRMPM) framework to realize the analysis method at the same time. Using this analysis method can have an overall understanding of the entire problem before in-depth study of the details. John [2] of RAND (the most important military-oriented comprehensive strategic research institution in the United States) proposed the hybrid, interactive, multiple-attribute, exploratory process (HIMAX). It is an operational effectiveness assessment framework that can comprehensively process expert experience data and simulation result data, and integrate fuzzy mathematics and analytic hierarchy process. Bassham et al. [3] proposed an effectiveness assessment framework for the automatic target recognition system (ATRCS) from the perspective of operational simulation experiments and assessment experts to establish the assessment model of ATRCS to complete the assessment of the effectiveness of ATRCS. Hootman et al. [4] proposed an efficiency analysis and decision support framework for the design and implementation of ships. Lee et al. [5] pointed out that it is difficult to determine the measurement of operational effectiveness in network centric warfare (NCW). For network centric warfare, the meta-network was used to express the relationship between weapons and equipment, and the multiplication of probabilities is used to obtain the measurement of operational effectiveness. Grzesik [6] used fuzzy theory to evaluate the effectiveness of aircraft systems and obtained the relationship between flight speed, airdrop height, attack angle and effectiveness through computer simulation. With the widespread application of big data and intelligent algorithms, Liu [7] and Li et al. [8] applied nontraditional research methods such as intelligent learning algorithms (neural networks, support vector machines) to equipment effectiveness assessment, providing a method for effectiveness assessment in new ways. Xu Jun [9] provided a stability analysis method of time-delay recurrent neural network based on the M-matrix method of the Lyapunov functional method. Wang et al. [10] used particle swarm, Elman, and wavelet neural network for target threat assessment, and improved the accuracy of efficiency assessment through network optimization. In order to further improve the convergence and robustness of the CPSOSQP algorithm, Xu Wenxing et al. [11] proposed two improved chaotic particle swarm optimization algorithms based on SQP global search. Felipe Petroski et al. [12] studied the weights of a DNN with a simple, gradient-free, population-based genetic algorithm (GA) and it performed well on hard deep RL problems, including Atari and humanoid locomotion. The Deep GA successfully evolved networks with over four million free parameters, the largest neural networks ever evolved with a traditional evolutionary algorithm. Compared with the error gradient in traditional deep learning, Joel Lehman et al. [13] proposed a solution by introducing a family of safe mutation (SM) operators that aim within the mutation operator itself to find a degree of change that does not alter network behavior too much, but still facilitates exploration.

Capability can be used to reflect the ability of equipment to perform missions under some mission requirements. This capability is determined by the performance of the equipment subsystems and the integration between the equipment subsystems [14,15]. Therefore, by analyzing and evaluating the capabilities of equipment systems, it is possible to determine the key indexes and weaknesses of the equipment system, so as to point out the equipment requirements and development direction, and provide a scientific basis for equipment construction.

**Figure 1:**
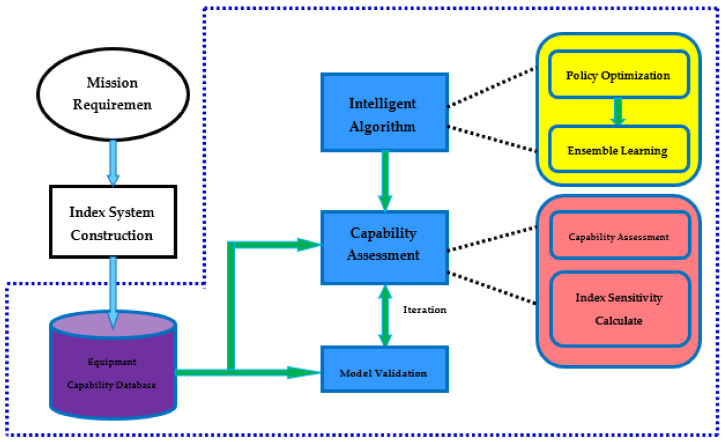
Capability assessment workflow.

The research work process of equipment capability assessment is shown in Figure 1. Taking mission requirements as the traction, we build a global index system. We then establish a database based on equipment indexes and equipment capabilities, that is, the Equipment Capability database in the figure, and provide data for capability assessment. Based on neural network, through different network policy optimization methods, the improved neural network algorithms obtained is ensemble learning, and the method is to find solutions to the generalization performance and local minima in neural network learning. On the basis of intelligent algorithm research, with the support of equipment indexes and capability database, an equipment capability assessment model is established, that is, the Capability Assessment in the figure. Through equipment capability estimation and data verification, the model is iteratively verified to solve the accuracy and stability of the model capability assessment. Taking the equipment capability assessment as the research object, the study of general equipment assessment methods provides support for the entire process, from equipment demonstration to operational use, and has significant scientific significance and application value. The focus of this paper is workflow shown in the blue dotted box of the figure.

Intelligent algorithms such as neural networks have achieved certain results in system assessment. However, it must be pointed out that most of the assessment methods focus on traditional methods and their improvements. The traditional assessment methods have their limitations: First, one cannot have a deep insight into the factors that affect effectiveness and the weight of these factors on effectiveness. Second, it is impossible to evaluate the rationality and typicality of the effectiveness assessment results under uncertain conditions. With the increase in the complexity of modern equipment systems, taking into account the dynamic nonlinear characteristics of complex equipment, intelligent algorithms such as artificial neural network optimization are suitable for dealing with complex nonlinear problems [16,17].

Based on the construction of the capability index system, this paper proposes an ensemble learning algorithm for policy optimization, which is used to evaluate capabilities, and solves the problems of neural network overfitting and global optimization. Finally, we use equipment capabilities to train the network, and evaluate the accuracy and stability of the algorithm through capability assessment. At the same time, through index sensitivity calculation, the sensitivity analysis of key indexes on equipment performance is realized. Therefore, the main contributions of this paper follow:

(1) The development of the ELPONN algorithm;
(2) The ELPONN algorithm is applied to the capability assessment field;
(3) Calculation and analysis of the sensitivity of equipment capability index.

Concerning the rest of this paper, we introduce neural networks and parameter optimization in Section 2. Section 3 describes policy optimization. Section 4 describes ensemble learning. Section 5 describes index sensitivity. Section 6 introduces the experiment and analysis. The conclusions are given in Section 7.

## 2. Neural Network and Parameter Optimization

Neural network is a widely parallel interconnected network composed of adaptable simple units whose organization can simulate the interactive response of biological nervous system to real-world objects. Given the training data set, the weight $w_{i}\left(i=1,2,\ldots ,n\right)$ and the threshold θ can be obtained through learning. The threshold *θ* can be regarded as the connection weight $w_{n+1}$ corresponding to a “dummy node” with a fixed input of −1.0. In this way, the learning of the weight and the threshold is unified into the learning of the weight. The perceptual learning rules are very simple. For the training example (*x*, *y*), if the current output of the perceptron is $\hat{y}$, the network weight will be adjusted as follows:

(1) $$w_{i}\leftarrow w_{i}+\Delta w_{i}$$

(2) $$\Delta w_{i}=\eta \left(y-\hat{y}\right)x_{i}$$

where $\eta \in \left(0,1\right)$ denotes the learning rate. If the network predicts the training example (*x, y*) correctly, that is $\hat{y}=y$, the network will not change, otherwise the weight will be adjusted according to the degree of error.

When trying to train multi-layer networks, simple network learning rules are obviously not enough, and more powerful learning algorithms are needed. The error back propagation (*BP*) algorithm is one of the best neural network learning algorithms.

### 2.1. BP Neural Network

*BP* neural network was proposed by a group of scientists led by Rumelhart and McCelland in 1986 [18] It is a multi-layer feed-forward network trained by the error back propagation algorithm and is currently one of the most widely used neural network models. The topological structure of the *BP* neural network model includes an input layer, a hidden layer, and an output layer. The dimensions of the input vector and output vector determine the number of hidden layers of the network and the number of neurons in each hidden layer. The BP neural network generally uses a secondary network.

As a supervised learning algorithm, the main idea of the BP algorithm is to input training samples and use the error back propagation algorithm to repeatedly train and adjust the network weights and thresholds, minimizing the deviation between the expected vector and creating an output vector that is as small as possible. The training ends when the error of the output layer meets the end condition, and the network weights and thresholds are saved.

#### 2.1.1. Network and Structure

**Figure 2:**
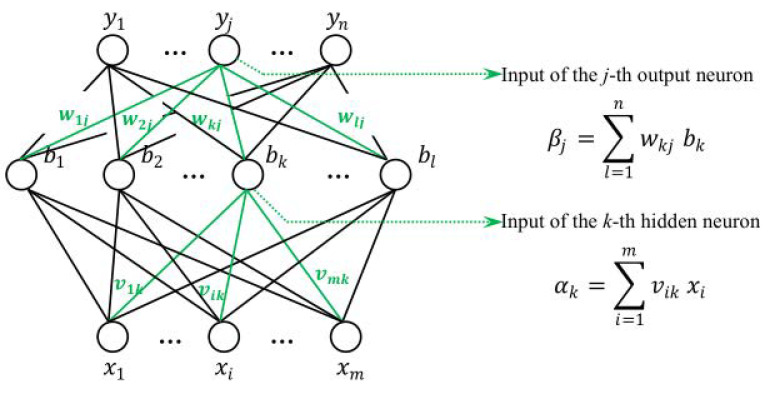
Structure of BP neural network.

Given training set $D=\left\{\left(x_{1},y_{1}\right),\left(x_{2},y_{2}\right),\ldots ,\left(x_{m},y_{m}\right)\right\},x_{i}\in {\mathbb{R}}^{d},y_{i}\in {\mathbb{R}}^{l},x_{i}\in {\mathbb{R}}^{d},y_{i}\in {\mathbb{R}}^{l}$; that is, the input sample is described by m attributes and the output an n-dimensional real-valued vector. Figure 2 shows a multi-layer feed-forward network structure with m input neurons, n output neurons, and one hidden layer neuron. The threshold of the *j*-th neuron in the output layer is represented by $\theta_{j}$, and the threshold of the *k*-th neuron in the hidden layer is represented by $\gamma_{k}$. The connection weight between the i-th neuron in the input layer and the *k*-th neuron in the hidden layer is $v_{ik}$, and the connection weight between the *k*-th neuron in the hidden layer and the *j*-th neuron in the output layer is $w_{kj}$. The input received by the *k*-th neuron in the hidden layer is $\alpha_{k}=\sum_{i=1}^{m}v_{ik}x_{i}$, and the input received by the *j*-th neuron in the output layer is $\beta_{j}=\sum_{l=1}^{n}w_{kj}b_{k}$, where $b_{k}$ is the output of the *k*-th neuron in the hidden layer [19].

For the training set $\left(x_{k},y_{k}\right)$, suppose the output of the neural network is ${\hat{y}}_{k}={\hat{y}}_{1}^{k},{\hat{y}}_{2}^{k},\ldots ,{\hat{y}}_{l}^{k}$:

(3) $${\hat{y}}_{j}^{k}=f\left(\beta_{j}-\theta_{j}\right)$$

Then the mean square error of the network on $\left(x_{k},y_{k}\right)$ is

(4) $$E_{k}=\frac{1}{2}\sum_{j=1}^{l}{\left({\hat{y}}_{j}^{k}-y_{j}^{k}\right)}^{2}$$

The BP algorithm is based on the gradient descent policy, which adjusts the parameters in the negative gradient direction of the target. For the current error $E_{k}$, given the learning rate η, there is

(5) $$\Delta w_{hj}=-\eta \frac{\partial E_{k}}{\partial w_{hj}}$$

Assuming that the hidden layer and output layer neurons use the tangent function sigmoid, according to the error derivation formula, the update formula for the connection weight $\omega_{hj}$ and the threshold $\theta_{j}$ in the BP algorithm can be obtained

(6) $$\Delta w_{hj}=\eta g_{j}b_{h}$$

(7) $$\Delta \theta_{j}=-\eta g_{j}$$

where

(8) $$g_{j}={\hat{y}}_{j}^{k}\left(1-{\hat{y}}_{j}^{k}\right)\left(y_{j}^{k}-{\hat{y}}_{j}^{k}\right)$$

Similarly available

(9) $$\Delta v_{ih}=\eta e_{h}x_{i}$$

(10) $$\Delta \gamma_{h}=-\eta e_{h}$$

where

(11) $$e_{h}=b_{h}\left(1-b_{h}\right)\overset{l}{\underset{j=1}{\sum }}w_{hj}g_{j}$$

The workflow of BP algorithm is shown in Algorithm 1. The input of the training sample to the input layer neurons is provided first, and then forward the signal layer-by-layer until the output layer results are generated. Then calculate the error of the output layer, and then propagate the error back to the hidden layer neurons. Finally, according to the hidden layer neurons, the error of the element is used to adjust the connection weight and threshold. This iterative process is repeated until certain stopping conditions are reached.
**Algorithm 1. Error Back Propagation Algorithm** Input: Training set D = ${\left\{\left(x_{k},y_{k}\right)\right\}}_{k=1}^{m}$; Learning rate η. Process: 1: Randomly initialize all connection weights and thresholds in the network within the range of (0,1) 2: repeat 3: for all $\left(x_{k},y_{k}\right)\in D\;do$ 4: Calculate the output ${\hat{y}}_{k}$ of the current sample according to the current parameters and formula (3); 5: Calculate the gradient term $g_{j}$ of the output layer neuron according to Formula (8); 6: According to Formula (11), calculate the gradient term $e_{h}$ of the output layer neuron; 7: According to Equations (6), (7), (9), (10), update the connection weights $w_{hj}$, $v_{ih}$ and the threshold $\theta_{j}$, $\gamma_{h}$; 8: end for 9: until stop condition reached Output: Multi-layer feed forward neural network with connection weight and threshold determined

#### 2.1.2. Anti-Overfitting

Due to the powerful representation ability, the BP neural network often has an overfitting problem. That is, its training error continues to decrease, but the test error may increase. There are two policies that can be used to alleviate the overfitting problem of BP network.

The first policy is “early stopping”: divide the data into a training set and a test set. The training set is used to calculate gradients and update connection weights and thresholds, and the validation set is used to estimate the error. If the training set error decreases but the test set error increases, stop training, and return the connection weight and threshold with the smallest test set error.

The second policy is “regularization”. The basic idea is to add a part of the error objective function to describe the complexity of the network, such as the square sum of the connection weights and thresholds. Let $E_{k}$ denote the error on the *k*-th training example and $w_{i}$ denote the connection weight and threshold. The error objective function is then

(12) $$E=\lambda \frac{1}{m}\sum_{k=1}^{m}E_{k}+\left(1-\lambda \right)\sum_{i}w_{i}^{2}$$

where $\lambda \in \left(0,1\right)$ is used to compromise the empirical error and network complexity, which is often estimated by cross-validation.

This paper improves the “early stopping” policy to prevent the algorithm from overfitting. The basic method follows: Pre-store the overall average error of the test set after five trainings and follow with training assessment. If the overall average error is less than all the pre-stored five-training error, replace the largest error in the pre-stored errors, and continue training estimation. If the overall average error is greater than the remaining five errors, stop the training assessment. The one with the smallest error among the remaining five errors is taken as the established network and used for operational aircraft capability assessment.

### 2.2. Parameter Optimization

If E is used to represent the error of the neural network on the training set, it is obviously a function of the connection weight *w* and the threshold *θ*. At this time, the training process of the neural network can be regarded as a parameter optimization process. That is, in the parameter space, a set of optimal parameters is found to make E the smallest.

There are usually two types of “optimum” involved: global minimum and local minimum. For $w^{*}$ and $\theta^{*}$, if ε>0 makes

(13) $$\forall \left(w;\theta \right)\in \{\left(w;\theta \right)|∥\left(w;\theta \right)-\left(w^{*};\theta^{*}\right)∥\leq \varepsilon$$

$E\left(w;\theta \right)\geq E\left(w^{*};\theta^{*}\right)$ exists, then $\left(w^{*};\theta^{*}\right)$ is a local minimum solution. If for any $\left(w;\theta \right)$ in the parameter space, $E\left(w;\theta \right)\geq E\left(w^{*};\theta^{*}\right)$ exists, then $\left(w^{*};\theta^{*}\right)$ is the global minimum solution. Intuitively, the local minimum solution is a point in the parameter space, and the error function value of its neighboring points is not less than the function value of the point. The global minimum solution refers to the error function value of all points in the parameter space that are not less than the error function value of the point. The corresponding $E\left(w^{*};\theta^{*}\right)$ is called the local minimum and the global minimum of the error function, respectively.

In real engineering, we often use the following policies to try to “jump out” from the local minima, and thus get closer to the global minimum:

(1) Initialize multiple neural networks with multiple sets of different parameter values and, after training according to the standard method, take the solution with the smallest error as the final parameter. This is equivalent to starting a search from multiple different initial points, so that it may fall into a different local minimum from which it is possible to obtain a result closer to the global minimum.
(2) Use the “simulated annealing” technique. Simulated annealing accepts a worse result than the current solution with a certain probability at each step, which helps to “jump out” of the local minimum. In each iteration process, the probability of accepting the “suboptimal solution” gradually decreases over time to ensure the stability of the algorithm.
(3) Use stochastic gradient descent. Unlike the standard gradient descent method that accurately calculates the gradient, the stochastic gradient descent method adds random factors when calculating the gradient. Therefore, even if it falls into a local minimum, its calculated gradient may still be nonzero, so there is a chance to jump out of the local minimum and continue searching.

In addition, genetic algorithms, particle swarm algorithms, firefly algorithms, bat algorithms, and cuckoo search algorithms are also commonly used to train neural networks to better approximate the global minimum.

## 3. Policy Optimization

Aiming at the structural and algorithm characteristics of the neural network, this paper optimizes the neural network from two aspects, search policy and gradient policy, so that the parameter optimization is close to the global minimum.

### 3.1. Search Policy

Typical methods for parameter optimization include genetic algorithms, particle swarm algorithms, firefly algorithms, bat algorithms, cuckoo search algorithms, etc. Among them, the genetic algorithm is the practice of simulating biological genetic inheritance. After the initial group is formed by coding, certain operations are imposed on individuals in the group according to their environmental fitness, so as to realize the evolution process of survival of the fittest, gradually optimizing the solution of search problems and approximating the optimal solution.

Starting from the update policy of gene fitness (reward) in the genetic algorithm, we study how to change the update policy to realize the traversal of the network weight space, so as to realize the global optimal policy. The genetic algorithm (*GA*) has evolved a population *P* of *N* individuals. In each generation, each $\theta_{i}$ is evaluated to produce a fitness value (also called reward) $F(\theta_{i})$. We use truncated selection for *GA* variation, where the top T individual becomes the parent of the next generation [12]. In order to produce the next generation, the following process is repeated *N*-1 times: the parent is randomly selected and replaced, and is abruptly changed by applying Gaussian noise to the parameter vector, where *ϵ*~*N*(0,1), the appropriate value of *σ* is determined by the experience of each experiment. The Nth individual is an unmodified copy of the best individual from the previous generation, called elitism. *GAs* often involve crossover (that is, combining parameters from multiple parents to produce offspring) and mutation, and then evaluating new populations. This process is repeated for *G* generations, or until some other stopping criteria are met. The genetic algorithm in Algorithm 2 outlines pseudo-code for this approach.
**Algorithm 2. Genetic Algorithm*** Input: mutation power σ, population size N, number of* 
*selected individuals T, policy initialization routine φ.* 
*for g = 1, 2, ..., G generations do* 
*for i = 1, ..., N in next generation’s population do* 
*if g = 1 then* 
$p_{i}^{g}$
*= φ(N(0,1)) {initialize random DNN}* 
$F_{i}^{g}$
$=F(p_{i}^{g}$
*) {assess its fitness}* 
*else* 
*if i = 1 then* 
$p_{i}^{g}\;$
$=p_{i}^{g-1}$
$;\;F_{i}^{g}=F_{i}^{g-1}$
*{copy the elite}* 
*else* 
*k = uniform Random (*1*,T) {select parent}* 
Sample ϵ
*~ N(0,I)* 
$p_{i}^{g}$
$=p_{i}^{g-1}+\sigma \epsilon$
*{mutate parent}* 
$F_{i}^{g}$
$=F(p_{i}^{g}$
*) {assess its fitness}* 
$\mathrm{Sort}\;p^{g}$
$\mathrm{and}\;F^{g}$
$\;\mathrm{with}\;\mathrm{descending}\;\mathrm{order}\;\mathrm{by}\;F^{g}$ 
$\mathrm{Return}:\mathrm{highest}\;\mathrm{performing}\;\mathrm{policy},\;p_{1}^{g}$


Although the *BP* neural network is the most widely used algorithm in the artificial neural network, it also has some shortcomings, such as a convergence speed that is too slow, the convergence to the global minimum point cannot be guaranteed, and the network structure is not easy to determine. In addition, the choice of network structure and initial connection weights and thresholds has a great impact on network performance, but it cannot be accurately obtained. For these problems, genetic algorithms can be used to optimize the BP neural network.

**Figure 3:**
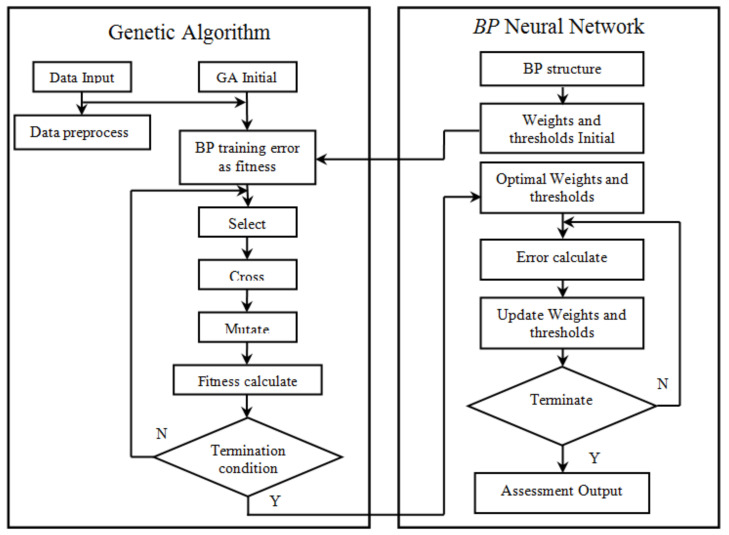
Optimization of BP neural network flow by the genetic algorithm.

The genetic algorithm optimizes the *BP* network algorithm flow as shown in Figure 3. Genetic algorithm optimization BP network is mainly divided into BP network structure determination, genetic algorithm optimization and threshold, and BP network training and prediction [20]. Among them, the topological structure of the BP network is determined by the number of input and output parameters of the sample, so that the number of optimization parameters of the genetic algorithm can be determined. The code length of the population individual can also be determined. Because genetic algorithm optimization parameters are the initial value and threshold of the BP network, as long as the network structure is known, the number of weights and thresholds will be known. The weights and thresholds of neural networks are generally randomly initialized to random numbers in the interval (−0.5,0.5). This initialization parameter has a great impact on network training, but it cannot be accurately obtained. For the same initial weights and thresholds, the training results are the same, and the genetic algorithm is introduced to optimize the best initial value and threshold.

### 3.2. Gradient Policy

Neural network is a deep learning framework, and the basic principle is a gradient descent mechanism. When using gradient descent, the flat area of the error surface will make the error decrease slowly, the adjustment time will be longer, the number of iterations will increase, and the convergence speed will be affected. Further, the multiple minima of the error surface will cause the network training to fall into a local minimum, thus making the network training unable to converge to the given error. These two problems are inherent shortcomings of the BP network standard algorithm. This paper optimizes the BP neural network from two aspects: stochastic gradient descent and speedup training.

#### 3.2.1. Stochastic Gradient Descent

Gradient-based search is the most widely used parameter optimization method. In this type of method, we start from some initial solutions and iteratively find the optimal parameters. In each iteration step, we first calculate the gradient of the error function at the current point and then determine the search direction according to the gradient. For example, since the negative gradient direction is the direction in which the function value drops the fastest, the gradient descent method is to search for the optimal solution along the negative gradient direction. If the gradient of the error function at the current point is zero, the local minimum has been reached, the update amount will be zero, and the iterative update of the parameters will stop. Obviously, if the error function has only one local minimum, then the local minima found at this time is the global minimum. However, if the error function has multiple local minima, there is no guarantee that the solution found is the global minima. For the latter case, we believe that the parameter optimization falls into a local minimum.

Unlike the standard gradient descent method that accurately calculates the gradient, the stochastic gradient descent method adds random factors when calculating the gradient. Each time the gradient is updated, m samples are used to add a random amount to adjust *θ*. Although the loss function obtained in each iteration step is not in the direction of the global optimum, the larger overall direction is the solution to the global optimum, and the final result is often close to the global optimum. Therefore, even if it falls into a local minimum, its calculated gradient may still be nonzero, so there is a chance to jump out of the local minimum and continue searching.

#### 3.2.2. Speedup Training

Neural network changes parameters along the negative gradient direction and sets a fixed learning rate to make the network learn. In order to improve the speed of network convergence, the study adopts the additional momentum method and the method of adapting the learning rate by parameter. Among them, the momentum update method improves the learning factor update policy. The parameter-by-parameter adaptive learning rate method can adapt to the method of adjusting the learning rate, and even the parameter-by-parameter adaptive learning rate adjustment, including methods such as Adagrad, RMSprop, and Adam.

Additional momentum method

BP neural network uses the gradient correction method as the learning algorithm of weights and thresholds. The weights and thresholds are corrected from the negative gradient direction of the network prediction error, without considering the accumulation of previous experience, and the learning process converges slowly. For this problem, the additional momentum method can be used to solve the problem [21]. The weight learning formula with additional momentum is

(14) $$w\left(k\right)=w\left(k-1\right)+\Delta w\left(k\right)+\alpha \left[w\left(k-1\right)-w\left(k-2\right)\right]$$

where $w\left(k\right),w\left(k-1\right),\;\mathrm{and}\;\;\;w\left(k-2\right)$ denote the weights at k, k−1, and k−2, respectively, and α is the momentum learning rate.

The value of BP neural network learning rate η is between (0,1). The larger the learning rate η, the greater the modification of the weight and the faster the network learning speed. However, a learning rate η that is too large will make the weight learning process oscillate, and a learning rate that is too small will cause the network to converge too slowly, while the weights are difficult to stabilize. The variable learning rate method means that the learning rate η is relatively large in the early stage of *BP* neural network evolution, and the network converges quickly. With the progress of the learning process, the learning rate continues to decrease and the network tends to stabilize. The variable learning rate calculation formula is

(15) $$\eta \left(k\right)=\eta_{max}-k\left(\eta_{max}-\eta_{min}\right)/K_{max}$$

In the formula, $\eta_{max}$ is the maximum learning rate, $\eta_{min}$ is the minimum learning rate, $K_{max}$ is the maximum number of iterations, and *K* is the current number of iterations.

Parameter-by-parameter adaptation method

The traditional parameter w is updated by accumulating the original w and a negative learning rate multiplied by the correction value *dx*, the method is *w*+ = *Learning rate* × *dx*. This kind of learning method will make the updating policy tortuous. Thus, to accelerate network calculations, we try to use some improved methods of neural network parameter research to apply them to the intelligent assessment of equipment capabilities. Common methods and mathematical expressions are shown in Table 1.

In the parameter-by-parameter adaptive learning rate method, the basic idea is to adjust the learning rate adaptively, or even to adjust the learning rate parameter by parameter. Among them, Adagrad receives the weight update effect if the high gradient value is weakened, and the update effect of weight receiving low gradient value will be enhanced. RMSprop is based on the Adagrad method, using a gradient-squared moving average. The Adam method is the RMSprop momentum version. Through the research of this method, it is applied to the neural network model of operational aircraft capability assessment. Starting from the learning efficiency of the neural network, the learning factor update method is used to realize the acceleration of the neural network training. Through the research on the momentum update and the parameter-by-parameter adaptive learning rate method, the key technology of learning factor update is broken through, and the neural network training acceleration method is realized.

## 4. Ensemble Learning

Ensemble learning completes the learning task by constructing and combining multiple learners, which is sometimes called a multi-classifier system.

### 4.1. Individual and Ensemble

The general structure of ensemble learning consists of generating a set of “individual learners” and then using a certain policy to ensemble them. Individual learners are usually generated from training data by an existing learning algorithm, such as the C4.5 decision tree algorithm, BP neural network algorithm, and others. At this time, the ensemble contains only the same type of individual learners. For example, “decision tree ensemble” is all decision trees and “neural network ensemble” is all neural networks. Such an ensemble is “homogeneous”.

The individual learner in a homogeneous ensemble is also called “base learner”, and the corresponding learning algorithm is called “base learning algorithm”. The ensemble can also include different types of individual learners, such as a simultaneous decision tree and a neural network. Such an ensemble is “heterogeneous”. The individual learners in a heterogeneous ensemble are generated by different learning algorithms, and there is no base learning algorithm at this time. The corresponding individual learners are generally not called base learners, but are usually called “component learners”.

### 4.2. AdaBoosting

According to the generation method of individual learners, the current ensemble learning methods can be roughly divided into two categories. The first is the serialization method, which must be generated serially because of the strong dependence between individual learners. The second is the parallelization method that can generate simultaneously without strong dependence among individual learners. The former is represented by Boosting, and the latter is represented by Bagging and “Random Forest”. This paper studies the use of the Boosting ensemble learning algorithm.

The AdaBoost algorithm [14] is a representative algorithm of the Boosting family. AdaBoost is an iterative algorithm. Its core idea is that the strong classifier used for decision-making is composed of different weak classifiers, and these weak classifiers are trained with the same training set. The different training sets in the Adaboost algorithm are achieved by adjusting the weights corresponding to each sample. At the beginning, the weight corresponding to each sample is the same, and a weak classifier is trained under this sample distribution. For samples with incorrect predictions, their corresponding weights are increased. For samples that are classified correctly, reduce their weights, so that the incorrectly classified samples are highlighted and a new sample distribution is obtained. Under the new sample distribution, the weak classifier is trained again, and so on; after T cycles, T weak classifiers are obtained, and these T weak classifiers are superimposed according to a certain weight to obtain the final strong classifier.

The AdaBoost algorithm uses a linear combination of base learners:

(16) $$H\left(x\right)=\sum_{t=1}^{T}\alpha_{t}h_{t}\left(x\right)$$

To minimize the exponential loss function:

(17) $$l_{exp}\left(H|D\right)=E_{x\sim D}\left[e^{-f\left(x\right)H\left(x\right)}\right]$$

In the AdaBoost algorithm, the first classifier $h_{1}$ is obtained by directly applying the base learning algorithm to the initial distribution. After that, $h_{t}$ and $\alpha_{t}$ are generated iteratively. After the base classifier $h_{t}$ is generated based on the distribution $D_{t}$, the weight of the base classifier $\alpha_{t}$ should make $\alpha_{t}h_{t}$ minimize the exponential loss function:

(18) $$l_{exp}\left(\alpha_{t}h_{t}|D_{t}\right)=E_{x\sim D_{t}}\left[e^{-f\left(x\right)\alpha_{t}h_{t}\left(x\right)}\right]\;=E_{x\sim D_{t}}[e^{-\alpha_{t}}Ⅱ\left(f\left(x\right)=h_{t}\left(x\right)\right)+e^{\alpha_{t}}Ⅱ\left(f\left(x\right)\neq h_{t}\left(x\right)\right)]\;=e^{-\alpha_{t}}\left(1-\epsilon_{t}\right)+e^{\alpha_{t}}\epsilon_{t}$$

where $\epsilon_{t}=P_{x\sim D_{t}}\left(f\left(x\right)\neq h_{t}\left(x\right)\right)$. Consider the derivative of the exponential loss function:

(19) $$\frac{\partial l_{exp}\left(\alpha_{t}h_{t}|D_{t}\right)}{\partial \alpha_{t}}=-e^{-\alpha_{t}}\left(1-\epsilon_{t}\right)+e^{\alpha_{t}}\epsilon_{t}$$

Let Formula (19) be zero, and then solved

(20) $$\alpha_{t}=\frac{1}{2}\ln\left(\frac{1-\epsilon_{t}}{\epsilon_{t}}\right)$$

Equation (20) is the classifier weight update formula of the Adaboost algorithm.

The weak classifier is trained based on the distribution $D_{t}$, and the error for $D_{t}$ should be less than 0.5. Considering the relationship between $D_{t}$ and $D_{t+1}$, there are

(21) $$D_{t+1}\left(x\right)=\frac{D_{x}e^{-f\left(x\right)H_{t}\left(x\right)}}{E_{x\sim D}\left[e^{-f\left(x\right)H_{t}\left(x\right)}\right]}\;=\frac{D_{x}e^{-f\left(x\right)H_{t-1}\left(x\right)}e^{-f\left(x\right)\alpha_{t}h_{t}\left(x\right)}}{E_{x\sim D}\left[e^{-f\left(x\right)H_{t}\left(x\right)}\right]}\;=D_{t}\left(x\right)e^{-f\left(x\right)\alpha_{t}h_{t}\left(x\right)}\frac{E_{x\sim D}\left[e^{-f\left(x\right)H_{t-1}\left(x\right)}\right]}{E_{x\sim D}\left[e^{-f\left(x\right)H_{t}\left(x\right)}\right]}$$

Equation (21) is the sample distribution update formula of the *Adaboost* algorithm.

Therefore, it can be seen from Equations (20) and (21) that we can obtain the *AdaBoost* algorithm from the perspective of iterative optimization of the exponential loss function based on the additive model, as shown in Algorithm 3.
**Algorithm 3. Adaboost Algorithm** Input: Training set $D={\left\{\left(x_{k},y_{k}\right)\right\}}_{k=1}^{m}$ Base learning algorithm L Number of training rounds T Process: 1: $D_{1}\left(x\right)=1/m$. 2: for *t* =1,2, …, *T* do 3: $h\left(t\right)=L\left(D,D_{t}\right)$; 4: $\epsilon_{t}=P_{x\sim D_{t}}\left(h_{t}\left(x\right)\neq f\left(x\right)\right)$; 5: if $\epsilon_{t}\;$> 0.5 then break 6: $\alpha_{t}=\frac{1}{2}\ln\left(\frac{1-\epsilon_{t}}{\epsilon_{t}}\right)$; 7: $D_{t+1}\left(x\right)=\frac{D_{t}\left(x\right)}{Z_{t}}\times \left\{\begin{array}{c} \exp\left(-\alpha_{t}\right),\;\begin{array}{cc} & if\;h_{t}\left(x\right)=f\left(x\right) \end{array}\\ \exp\left(\alpha_{t}\right),\;\begin{array}{cc} & if\;h_{t}\left(x\right)\neq f\left(x\right) \end{array} \end{array}\right.=\frac{D_{t}\left(x\right)\exp\left(-\alpha_{t}f\left(x\right)h_{t}\left(x\right)\right)}{Z_{t}}$ 8: end for Output: $H\left(x\right)=\mathrm{sign}\left(\sum_{t=1}^{T}\alpha_{t}h_{t}\left(x\right)\right)$


### 4.3. Policy Optimization Ensemble

Learner ensemble may bring benefits from three directions [19]: First, from a statistical point of view, since the hypothesis space of learning tasks is often large, there may be multiple hypotheses that achieve the same performance on the training set. At this time, if a single learner is used, it may lead to poor generalization performance due to miss election. Combining multiple learners will reduce this risk. Second, from a computational perspective, the learning algorithm tends to fall into a local minimum, the generalization performance corresponding to some local minima may be very bad, and the combination after multiple runs can reduce the risk of falling into the local minima. Third, from the perspective of representation, some learning task hypotheses may not be in the hypothesis space considered by the current learning algorithm. At this time, if a single learner is used, it is definitely invalid. By ensemble multiple learners, due to the expansion of the corresponding hypothesis space, it is possible to learn better assessment.

Assume that the ensemble contains T base learners $\left\{h_{1},h_{2},\ldots ,h_{T}\right\}$, where the output of $h_{i}$ on input *x* is $h_{i}\left(x\right)$. The combined policy by the weighted average method can be expressed as

(22) $$H\left(x\right)=\sum_{i=1}^{T}\omega_{i}h_{i}\left(x\right)\;$$

where $\omega_{i}$ is the weight of individual learner $h_{i}$, usually requiring $\omega_{i}\geq 0$*,* $\sum_{i=1}^{T}\omega_{i}=1$.

**Figure 4:**
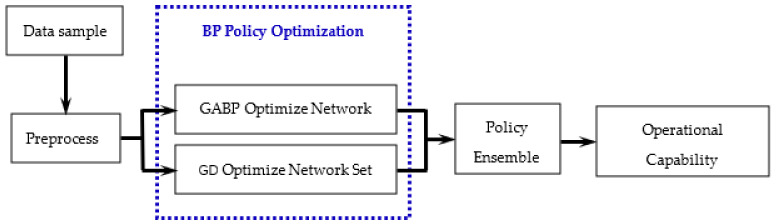
Capability assessment model based on ensemble learning of policy optimization.

In this paper, various algorithm agents are generated for different types of individual learners, and different optimization algorithms are combined into an ensemble learning algorithm by the weighted average method. Among them, each agent optimizes the BP neural network based on search policy and gradient policy. Use the ensemble method to construct the equipment capability assessment model, as shown in Figure 4.

## 5. Index Sensitivity

With the indexes of subequipment and system equipment, some performance index of subequipment can be expressed by physical formulas. The sensitivity of the index is perturbed by the perturbation method, that is, the corresponding change in the output result of the equipment capability after a small change in certain conditions. Explore the index analytical solution method by calculating and analyzing the absolute sensitivity and relative sensitivity of the index, so as to establish the correlation model between the sensitivity of the equipment index and the equipment capability. In most cases, it is difficult to establish a quantitative model relationship between performance index and equipment capabilities. Subjective judgment can be used to determine the spatial range of index that meet equipment capabilities, and the sensitivity calculation and analysis methods can be studied with reference to the discrete index method.

At the same time, in the equipment capability assessment, corresponding to the relationship between the index system and the equipment capability, the sensitivity of the index is analyzed. On the one hand, the relative importance of the current indexes can be analyzed, that is, which index is more sensitive to the improvement of the equipment capability. Analyze the main factors of the index system. On the other hand, it can be used as an objective basis for the optimization of the index system, by putting forward key indexes and achieving quantitative and objective assessment of equipment capabilities. In the analysis, the relationship between indexes is measured by defining absolute sensitivity and relative sensitivity. The absolute sensitivity refers to the perturbation analysis of each index relative to its own global scope. The relative sensitivity refers to the degree of influence of each index on the change of the total performance after the perturbation changes within the range of the current value, and the change of the total performance is also relative to the current range change.

At present, the most commonly used neural network algorithm proposes a connection method similar to human brain neurons to determine the adaptive, nonlinear, and parallel processing capabilities of the system, thereby simulating the system and estimating the output of the system. For the analysis of system changes, Salitelli et al. [22,23] proposed a parameter sensitivity model to study how the output changes of the mathematical model can be allocated quantitatively or qualitatively according to changes in the input source. Through the combination of neural network and sensitivity analysis, this paper uses the known input parameters of the system to realize the output prediction of operational aircraft capabilities. Simultaneously, it analyzes the relationship between the input and output of the system, so as to realize the purpose of analyzing the system by the mathematical model; establish an index space for operational aircraft capabilities, calculate the correlation model of index sensitivity to equipment capabilities, analyze the impact of index changes on equipment capabilities, and build an equipment index system. Sensitivity refers to the analysis of changes in equipment capabilities when there are disturbance changes in the actual index values. The purpose of sensitivity calculation and analysis is to understand the relative importance of the current indexes, that is, which index improvement is more sensitive to the improvement of the system’s capabilities. In the pre-research and demonstration, the main link of the index is grasped. The equipment index sensitivity is the equipment index analysis and important aspects of design. Equipment indexes are divided into discrete and continuous types. Based on the discrete interval of a certain index, such as the sensitivity calculation and analysis of the multi-dimensional space of the full index, it is obvious that the workload is large and the efficiency is low, and there is much information interference analysis. Therefore, through the index space optimization method, the multi-dimensional space area is searched and optimized, and the purpose is to achieve the effect of calculating all the required calculation areas with a small number of space area points.

### 5.1. Sensitivity Model

For an engineering system, studying the sensitivity of a certain index can determine the influence of the system on the output of the system when the index changes in the global scope under the normal state. This research method is also called the local sensitivity analysis method.

For a time-independent hydrostatic system, the system agenda can be expressed as

*y* = f(*x*) (23)

where *y = *(*y*_1_*, y*_2_*, …, y_n_*)*^T^* is the output variable of the system and *x* = (*x*_1_*, x*_2_*, …, x_m_*)*^T^* is the input variable of the system, namely the system index.

When the system input parameter *x* is slightly perturbed by Δx, the corresponding system output *y* can be expanded using Taylor’s formula:

(24) $$y\left(x+\Delta x\right)=y\left(x\right)+\overset{m}{\underset{j=1}{\sum }}\partial y/\partial x_{j}\Delta x_{i}+\frac{1}{2}\overset{m}{\underset{i=1}{\sum }}\overset{m}{\underset{j=1}{\sum }}\left(\frac{\partial^{2}y}{\partial x_{i}\partial x_{j}}\right)\Delta x_{i}\Delta x_{j}+\ldots$$

where the first-order partial differential $\partial y_{i}/\partial x_{j}$ is called the first-order sensitivity index, the second-order partial differential is called $\partial^{2}y_{i}/\partial x_{j}\partial x_{k}$ is called the second-order sensitivity index, and so on. In actual research work, because the first-order sensitivity index can more comprehensively reflect the trend and size of the system model with the input parameters at a certain point, it is often used as a local sensitivity analysis index, and the corresponding sensitivity matrix is

(25) $$S=\left(S_{ij}\right)=\left(\partial y_{i}/\partial x_{j}\right)$$

When the system is a single output model, the sensitivity of a certain parameter is

(26) $$S=\left(S_{i}\right)=\left(\partial y/\partial x_{i}\right)$$

### 5.2. Index Sensitivity Based on Neural Network

The index sensitivity study analyzes the influence of each index on the system output performance by calculating the influence factors of each index of the system on the system output, and then serves as an objective basis for the optimization of the index system, proposes key indexes, and realizes quantitative and objective assessment of equipment capabilities.

The BP neural network is a multi-layer forward neural network based on an error back propagation algorithm. The transfer function includes threshold function, linear function, logarithmic function, and tangent function. Among them, the logarithm and tangent function are the keys to realize the nonlinear mapping mechanism, which is also called the sigmoid conversion function. Since the sigmoid-type function is a differentiable function, it provides a mathematical theoretical guarantee for calculating the sensitivity matrix [24].

For a BP neural network with a hidden layer, if the output of the network is *Out* and the input of the network is *In*, the mapping relationship of the neural network is

(27) $$In_{1}\;=w_{1}\times \mathrm{In}+b_{1}\;\mathrm{Out}1_{\;}=f_{1}(In_{1})$$

(28) $$In_{2}\;=w_{2}\times \mathrm{Out}1_{\;}+b_{2}\;\mathrm{Out}=f_{2}(In_{2})$$

where *In*_1_ is the input vector of the hidden layer neuron and *In*_2_ is the input vector of the output layer neuron. The transfer function of the hidden layer selects the hyperbolic tangent function, as shown in Equation (29). The transfer function of the output layer is a linear function, as shown in Equation (30), and let *k* = 1.

(29) $$ut=\left(e^{in}-e^{-in}\right)/\left(e^{in}+e^{-in}\right)$$

(30) out=k×in

The sensitivity matrix of the network output *Out* to the network input *In* can be calculated by the following formula:

(31) $$\frac{\partial Out}{\partial In}=\frac{\partial Out}{\partial In_{2}}\frac{\partial In_{2}}{\partial Out_{1}}\frac{\partial Out_{1}}{\partial In_{1}}\frac{\partial In_{1}}{\partial In}=f_{2}^{'}\left(In_{2}\right)\times w_{2}\times f_{1}^{'}\left(In_{1}\right)\times w_{1}$$

Derivation of the hidden layer and output layer functions:

(32) $$f_{1}^{'}\left(x\right)=1-f_{1}^{2}\left(x\right)f_{2}^{'}\left(x\right)=1$$

Substituting Formula (32) into Formula (31):

(33) $$\frac{\partial Out}{\partial In}=w_{2}\times \left\{1-f_{1}^{2}\left(w_{1}\times In+b1\right)\right\}\times w_{1}$$

Equation (33) is the general expression for calculating the sensitivity matrix of the BP neural network with a hidden layer. After training the BP neural network with system data, the parameters $w_{1}$,$\;b_{1}$ and $w_{2}$ can be directly determined by the network structure, so that the sensitivity matrix can be calculated.

First, combined with the operational capabilities, analyze the performance space of equipment indexes; second, calculate the sensitivity of operational aircraft capabilities through optimized machine learning methods; third, generate the correlation between index sensitivity and equipment capabilities, and reduce the index space based on the assessment results, iterate repeatedly, and propose key indexes. The relationship between key indexes and operational aircraft capabilities is given, and an operational aircraft capability assessment method based on the sensitivity of key elements of the indexes is established to achieve quantitative and objective assessment of operational aircraft capabilities.

## 6. Experiments

### 6.1. Equipment Index

The equipment capability assessment gives the calculation index in the whole stage of the equipment process through the analysis of the equipment task to the capability demand. For a comprehensive and scientific assessment, it is crucial to establish a reasonable assessment index system. According to task requirements, the index system must be explored and analyzed, because it is difficult to establish analytical expressions between indexes and equipment capabilities, and the index system shows a multi-level system structure. This is done by using variance analysis, main component analysis, and branch boundary screening, on the basis of the guidance mode and general method of the index system.

According to the construction of the equipment index system, the index method is used to measure the capability against the seven main indexes of related equipment in this paper. They are mobility, firepower, ability to detect targets, control effectiveness, survivability, range, and electronic counter capability. The coefficients of electronic counter capability are estimated according to the coefficients of electronic counter equipment installed on aircraft. In the equipment capability assessment, 10 sets of data are used as test sample data to test the fitting performance of the network by randomly extracting from the database, and the remaining data are used as network training sample data for network training. The data are normalized and unified into dimensionless data in the interval (0,1).

### 6.2. Parameter Setting

The experiment in this paper runs in Matlab2012. The BP network parameters are 100 iterations and a learning rate of 0.1. The genetic algorithm parameters are set as follows: the number of iterations is 20, the population size is 10, the crossover selection probability is 0.2, and the mutation selection probability is 0.1.

In the ensemble learning network, each agent optimizes the *BP* neural network based on search policy and gradient policy. The policy optimization ensemble learning algorithm uses the weighted average method and the policy to construct the equipment capability assessment network.

### 6.3. Capacity Assessment

**Figure 5:**
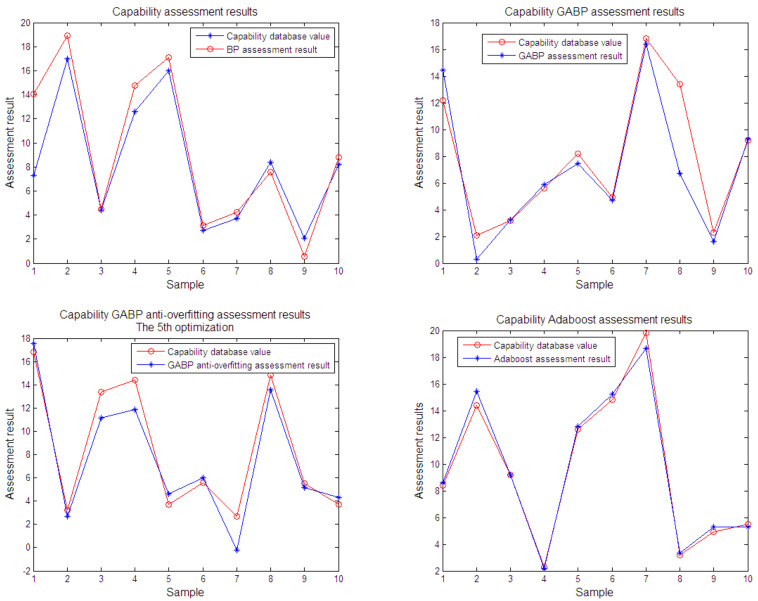
The curves of capability assessment uses BP, GABP, anti-overfitting (early stopping), and Adaboost (ELPONN) algorithm.

In order to directly reflect the effect of the algorithm on equipment capabilities assessment, the results of BP neural network, GABP optimized neural network, early-stopping algorithm and ELPONN are shown in Figure 5. In the figure, the blue curve represents the data in the database and the red curve shows the capability assessment curve. It can be seen from the figure that the assessment matches the equipment capability data to a certain degree, and the ELPONN method by *Adaboost* algorithm has the highest accuracy.

**Figure 6:**
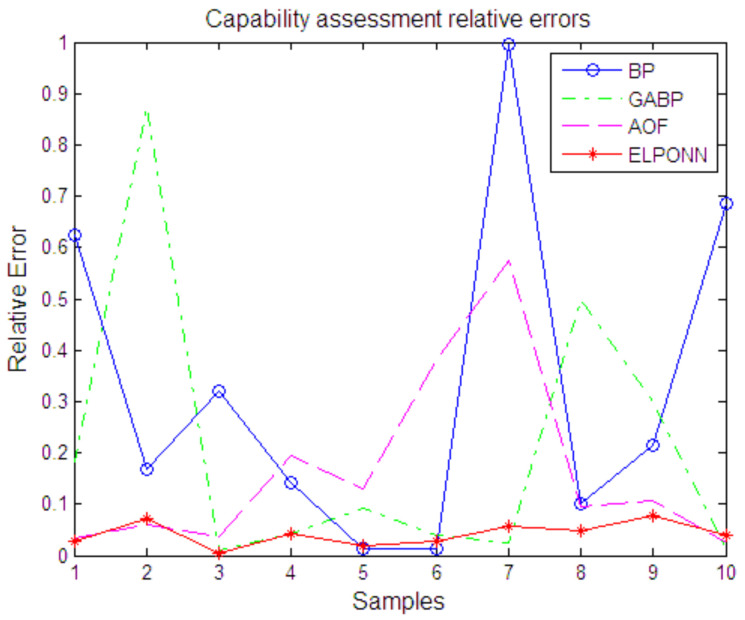
Capability assessment relative errors.

In order to compare the performance of the algorithm intuitively, the capability assessment relative errors by each algorithm are shown in Figure 6. The ELPONN is 1.89~7.13%, which is far better in accuracy than BP Network (1.3~99.4%), GABP (0.91~87.3%), or AOF (2.57~57.4%). At the same time, the ELPONN has higher stability than other methods.

The mean absolute errors (MAE) and mean relative errors (MRE) are as follows (from large to small): BP > GABP > anti-overfitting (early-stopping) > ELPONN, as shown in Table 2. This shows that the ELPONN algorithm has better performance.

### 6.4. Sensitivity Calculation and Analysis

Establishing an equipment index space, we calculate the correlation model of index sensitivity to equipment capabilities, analyze the impact of index changes on equipment capabilities, and build an equipment index system. Sensitivity refers to the analysis of changes in equipment capabilities when there are disturbance changes in the actual index values. The purpose of sensitivity calculation and analysis is to understand the relative importance of the current indexes. That is, the improvement of which index is more sensitive to the improvement of the capability of the system. The equipment index sensitivity is the equipment index analysis and important aspects of design. Equipment indexes are divided into discrete and continuous types. Based on the discrete interval of a certain index, such as the sensitivity calculation and analysis of the multi-dimensional space of the full index, it is obvious that the workload is large and the efficiency is low, and there is much information interference analysis. Based on the equipment capability database, this paper uses the ensemble learning based on policy optimization algorithm to learn the capabilities and calculate the index sensitivity.

**Figure 7:**
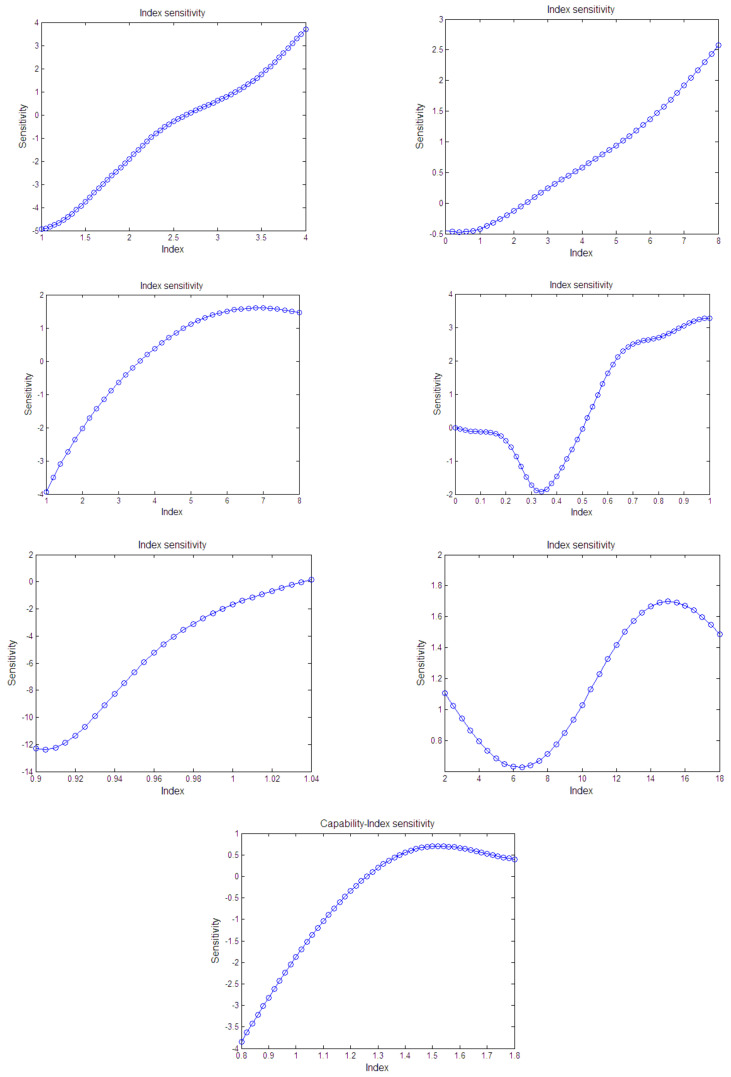
Sensitivity of capability index.

Figure 7 shows the sensitivity of the equipment capability index. It can be seen from the figure that some sensitivities increase with the index values, and some will be saturated. In addition, some indexes are more sensitive to the capability in the middle range.

## 7. Conclusions

According to the research requirements of equipment capability assessment, and aiming at the accuracy and stability problems encountered in the assessment of a large number, multiple types, and high complexity of the global index system, the neural network algorithm is optimized through the evolution of different strategies. We build an ensemble learning model of multi-intelligence algorithms. Based on the construction of the equipment capability index system, the ELPONN algorithm is used to assess equipment capabilities. This is accomplished in the following steps: optimize BP neural network through gradient policy and search policy, use the policy combination method for ensemble learning of optimized multi-intelligence network algorithm, assess equipment capabilities, and calculate index sensitivity. The assessment results show that *ELPONN* achieves better accuracy and stability requirements, and meets the requirements of capability assessment.

In the next step, we will further study ensemble learning algorithms such as neural networks, reinforcement learning, and evolutionary learning to solve the problem of intelligent algorithms in capability assessment.

## Figures and Tables

**Table 1:** Network parameter update algorithm.

Algorithm	Mathematical Expression	Algorithm Features
Momentum	*m = b*1 × *m − Learning rate* × *dx*	Introduce gradient descent inertia
	*w + m*	
AdaGrad	*v+ = dx^*2	Introduce false descent resistance
	*w+ = Learning rate* × *dx/*$\sqrt{v}$	
*RMSPROP*	*v = b*1 × *v+(*1 *− b*1*)* × *dx^*2	Introduce gradient descent inertia
	*w+ = −Learning rate* × *dx/*$\sqrt{v}$	and false descent resistance
*Adam*	*m = b*1 × *m + (*1 *− b*1*)* × *dx*	Introduce gradient descent inertia
	*v = b*2 × *v + (*1 *− b*2*)* × *dx^*2	and false descent resistance
	*w+ = −Learning rate* × *m/*$\sqrt{v}$	

**Table 2:** Assessment errors for different algorithms.

	BP	GABP	Early-Stopping	ELPONN
MAE	1.78	1.32	1.03	0.9
MRE	32.81%	20.75%	16.37%	4.10%

## Data Availability

Data sharing not applicable. No new data were created or analyzed in this study. Data sharing is not applicable to this article.
